# An optimal selection method of wells for secondary fracturing in a single coal seam and its application

**DOI:** 10.1038/s41598-022-10107-0

**Published:** 2022-04-12

**Authors:** Xuebin Tan, Xiaoming Ni, Zhongcheng Li, Zhiwen Xiong, Xiao Liu

**Affiliations:** 1 School of Energy Science & Engineering, Henan Polytechnic University, Jiaozuo, 454000 China; 2 Institute of Efficient Development and Utilization of Coal-Measure Gas, Henan Polytechnic University, Jiaozuo, 454000 China; 3 China United Coalbed Methane Corporation, Ltd, Beijing, 100011 China

**Keywords:** Geology, Engineering

## Abstract

At present, methods including mathematical modeling, physical or numerical simulation, and in-situ monitoring have been generally adopted to determine evaluation parameters for coalbed methane (CBM) wells for secondary fracturing. These conventional methods either entail many assumptions, or some parameters are difficult to obtain, resulting in a certain deviation between the evaluation results and reality, or the application cost is high, preventing the monitoring of each CBM well. In view of this, an evaluation index system for the gas production potential, effective length of cracks formed by fracturing, and supporting length of proppant in cracks was established based on the system theory. The evaluation indices were characterized through production data, such as logging, fracturing and drainage, which could avoid potential bias in evaluation when only considering a certain parameter and ensured accuracy and practicability of the evaluation parameters for each well. Principal component analysis (PCA) and the entropy weight method (EWM) were used to obtain weights of evaluation parameters, which avoided the contradiction of contributions of various parameters to optimal selection and the rationalized results. In this way, a method for step-wise optimal selection of wells for secondary fracturing integrating construction of evaluation parameters, determination of critical values, and entropy evaluation was proposed. The results of an evaluation of the Shizhuang South Block of Qinshui Basin (Shanxi Province, China) indicate that wells whose three evaluation indices are satisfied are most preferable; wells that only meet the effective length of cracks formed by fracturing or effective supporting length of proppant in cracks can be selected; wells which do not meet the gas production potential or all of the three parameters cannot be selected.

## Introduction

Whether secondary fracturing can be conducted in a single coal seam is mainly determined by parameters, such as the gas production potential, extension length of cracks formed by primary fracturing, and effective supporting length of proppant in cracks formed by primary fracturing^1–3^. At present, the gas production potential of coalbed methane (CBM) wells before fracturing has been generally characterized by the gas content in coal seams, the coal thickness, and reservoir pressure. However, the gas content in coal seams and reservoir pressure change over a period of drainage of CBM wells after primary fracturing. Therefore, these evaluation parameters cannot be accurately obtained at present, so that selection of wells for secondary fracturing based on these parameters may be sub-optimal in practice.

At present, methods, including mathematical modeling, physical or numerical simulation, and in-situ monitoring are used to evaluate fracturing for coal-bed methane (CBM) wells. Based on fracture mechanics, elasto-plastic mechanics, and other theories, engineers and scientists working on coal-bed methane projects have established mathematical models of fracture initiation and extension^4,5^. These mathematical models all entail certain assumptions, and some parameters are obtained from experience, resulting in deviations from reality.

The fracture morphology and length were studied through the experimental simulation of three-axis hydraulic fracturing, combined with fracture monitoring methods such as acoustic emission technology, CT scanning, and nuclear magnetic resonance, as well as the use of numerical simulation software such as Flac 3D, Ansys, and RFPA^2D^-Flow^6–8^. Physical simulation methods are generally cumbersome processes and mostly use similar materials. The simulated environment and in-situ differences may lead to significant differences between the results and reality, however, numerical simulation results are often able to lead to regular conclusions, and the specific guidance should be combined with field monitoring, experimental testing, and other methods.

In terms of field monitoring, the earth potential method, microseismic monitoring, and other techniques are often used to obtain the value of fracture extension length of a single fracture. This method is relatively expensive, and it is usually impossible to monitor each fracture well on any given project^9,10^.

The above research results provide reference for selecting wells for secondary fracturing in a single coal seam; because it is relatively difficult to determine these parameters, it is impossible to evaluate each well. In the meanwhile, due to the limitation of the methods for obtaining parameters, the evaluation values of some parameters are significantly different from the true values, which can also influence the accuracy of the evaluation to a certain extent.

From the perspective of system theory, an evaluation index system of wells for secondary fracturing in a single coal seam was constructed by making full use of in-situ data. This could avoid bias when only considering one evaluation parameter and ensured the accuracy and practicability of obtaining the evaluation parameters of each well. Critical values of each parameter were determined according to production data of more than 100 CBM wells in the Shizhuang South Block of Qinshui Basin. The weights of wells for secondary fracturing were determined through principal component analysis (PCA) and an entropy method and wells for secondary fracturing in the Shizhuang South Block were selected in a step-wise manner. The research aims to provide a method and reference for selecting wells for secondary fracturing in this area under similar geological conditions.

## Overview of geology and production in the study area

**Figure 1:**
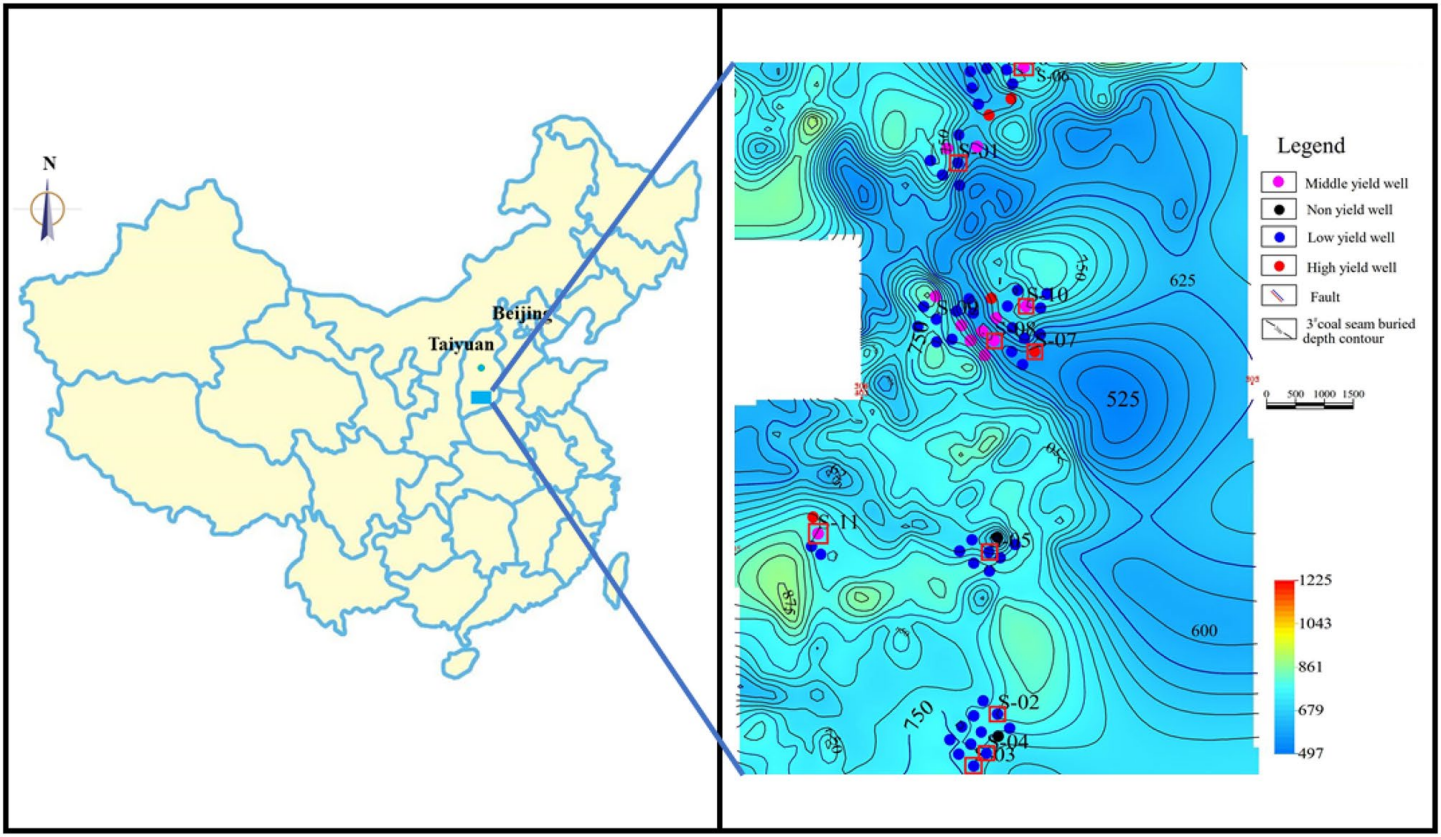
The average daily gas production in the study area. Map was drawn by DF-GVision4.0. URL link: http://www.gdfoil.com/Index/lists/catid/16.html.

Shizhuang South Block is located in the central-eastern region of the Qinshui Basin, in which the 3# coal seam is mainly mined. The 3# coal seam is generally buried 450 to 1000 m below ground and has a coal thickness of about 6 m and gas content of 8.11 to 21.51 m^3^/t. The coal structure is mainly composed of cataclastic coal and granular coal. At present, more than 800 production wells have been built in this block, but the gas production of more than 80% of wells does not exceed 1000 m^3^/d. The average daily gas production in the study area is illustrated in Fig. 1.

## Study methods

To ascertain whether secondary fracturing can be conducted in CBM wells, it is firstly necessary to meet the requirements for the gas production potential. Secondly, it is essential to evaluate the effective extension length of cracks formed by primary fracturing. Finally, it is required to evaluate the effective supporting length of proppant in cracks formed by primary fracturing. From these three aspects, an evaluation index system was constructed, and evaluation parameters were characterized by making full use of field production data derived from logging, fracturing, and drainage. Furthermore, critical values of evaluation parameters were determined. Based on PCA and an entropy method, weights of each evaluation parameter were obtained and a method for step-wise optimal selection of wells for secondary fracturing was proposed.

### Establishment of the evaluation index system

#### Evaluation parameters for the gas production potential

CBM wells with high gas production potential are a basis for secondary fracturing. On the premise of keeping other conditions constant, excessive daily water yield hinders the propagation of water pressure and makes it difficult to reduce the pressure in the coal seam in the single-phase water flow stage of on-site drainage. To render all wells comparable, the daily water yield under the unit pressure difference, namely the index of daily water yield, was used to reflect the difficulty of pressure propagation (albeit indirectly), so the index could be used as an evaluation parameter. Similarly, all other conditions being equal, the higher the gas content in the coal seam, the more the gas produced from the coal seam. The desorption pressure gradient refers to the change in the critical desorption pressure per unit length under gas migration. The larger the desorption pressure gradient, the stronger the driving force for gas migration after desorption, which is more conducive to gas production. The reservoir pressure gradient refers to variation in the reservoir pressure per unit length during fluid flow. As other conditions are unchanged, the larger the reservoir pressure gradient, the further the water flows. If gas can be desorbed, the larger the desorption area, the more the gas desorbed. These parameters can be used as parameters to evaluate the gas production potential.

1. Daily water yield
  According to the definition of the daily water yield, it is given by:
  1 $$W_{ri} = \lg \left( {\frac{{w_{r} }}{\Delta p}} \right)$$
  where, *W*_ri_, $w_{r}$, and $\Delta p$ represent the index of daily water yield, daily water yield (m^3^/t) under stable single-phase water flow, and pressure difference (MPa) in the stable period, respectively.
2. Gas content
  The gas content (*V*) was obtained by using the methods, including isothermal adsorption method and logging and testing^11,12^.
3. Desorption pressure gradient
  The desorption pressure could be calculated by the produced gas fluid level and casing pressure at the moment of gas production through drainage^13,14^, which is expressed as follows:
  2 $$P_{\lg } = \rho \times g \times (h_{m} - h_{l} ) \times 10^{ - 6} + p_{t}$$
  where, $P_{\lg }$, *h*_m_, and *h*_l_ represent the desorption pressure gradient (MPa/m), burial depth (m) of the coal seam, and the produced fluid level (m) at the moment of gas production, respectively; $\rho$ and *p*_*t*_ denote the density (g/cm^3^) of water and casing pressure (MPa) at the moment of gas production, respectively.
4. Reservoir pressure gradient
  In accordance with the initial producing fluid level upon CBM drainage, the reservoir pressure gradient could be calculated in combination with the burial depth of the coal seam, which is expressed as follows:
  3 $$P_{g} = \rho \times g \times (h_{m} - h_{c} ) \times 10^{ - 6} /h_{m}$$
  where, *P*_g_ and *h*_c_ denote the reservoir pressure gradient (MPa/m) and height (m) from the mouth of a well to the initial produced fluid level, respectively.

#### Evaluation parameters for the effective length of cracks formed by fracturing

When the effective length of cracks for fracturing is large, the gas production is greater. During fracturing, when the proportion of primary coal and cataclastic coal in the coal seam is below a certain value, the fracturing fluid will hardly flow into the primary coal and cataclastic coal during hydraulic fracturing, making the formation of effective cracks impossible. The so-called effective extension length for fracturing denotes the extension length of cracks formed by fracturing in primary coal and cataclastic coal. Within a certain range of coal thickness, the greater the proportion of primary coal and cataclastic coal in the total coal thickness, the larger the effective length of cracks formed by fracturing, so it can be used as one of the evaluation parameters. When there is no sand production and plugging during hydraulic fracturing, the range of fluctuation of the operating pressure is generally not excessive. A large range of fluctuation, on the one hand, may be induced by a high operating pressure at the beginning due to severe pollution near the wellbore. After breaking through the pollution zone, the operating pressure drops sharply, which may result in less effective pre-pad fluid and limited effective length of formed cracks. On the other hand, it may be because a large proportion of granular coal and mylonitic coal, which can cause the extrusion and puncture during fracturing and therefore leads to pressure fluctuations and a limited effective extension length of cracks formed by fracturing. Therefore, the effective length of cracks can be evaluated through the index of the operating pressure fluctuation. When the zone near a wellbore is severely polluted, the operating pressure at the initial stage of fracturing is high; after breaking through the pollution zone, the operating pressure suddenly decreases, and the amount of pre-pad fluid can be determined through the breakthrough point. The reservoir pollution index indicates the ratio of the amount of pre-pad fluid before sudden change in operating pressure to that afterwards, which is used to evaluate the effective length of the cracks.

1. Proportion of thickness of primary coal and cataclastic coal
  The thickness of coal with different structures was determined by logging and the proportion of thickness of primary coal and cataclastic coal was calculated as follows^15,16^:
  4 $$C_{sr} = \frac{{C_{pc} }}{{C_{t} }}$$
  where, *C*_*sr*_, *C*_*pc*_, and *C*_*t*_ represent the proportion of thickness of primary coal and cataclastic coal (dimensionless), thickness (m) of primary coal and cataclastic coal in the coal seam, and the total thickness (m) of the coal seam, respectively.
2. Index of the operating pressure fluctuation
  This index is given by:
  5 $$p_{s} = \sqrt {\frac{{\sum\nolimits_{i = 1}^{n} {(p_{i} - \overline{p} )^{2} } }}{n}}$$
  where, $p_{s}$, *p*_*i*_, and $\overline{p}$ denote the index of operating pressure fluctuation (dimensionless), operating pressure (MPa) at any time during fracturing of pre-pad fluid, and average operating pressure (MPa) in the fracturing period of pre-pad fluid, respectively.
3. Reservoir pollution index
  Based on the definition, the reservoir pollution index is expressed as follows:
  6 $$R_{pi} = \frac{{Q_{pb} }}{{Q_{pa} }}$$
  where, *R*_*pi*_, *Q*_*pb*_, and *Q*_*pa*_ denote the reservoir pollution index (dimensionless), amount (m^3^) of pre-pad fluid before the sudden change in operating pressure, and amount (m^3^) of pre-pad fluid thereafter, respectively.

#### Evaluation parameters for the effective supporting length of proppant in cracks

The effective supporting length of proppant in cracks represents the supporting length of proppant in the developed cracks. In the stable production of CBM wells, the daily water yield and daily gas production are quasi-stable. After eliminating interference from extraneous factors, when the daily water yield and daily gas production suddenly change, it indicates that the conductivity of the reservoirs has decreased. Combined with the drainage time, it can then be judged whether the boundary of the effective supporting length of proppant in cracks is reached. The indices of sudden changes of the daily water yield and daily gas production were employed to characterize the index.

1. Index of sudden change in daily water yield
  The index of sudden change in daily water yield indicates the ratio of the daily water yield of the previous day to that of the next day under normal drainage and production in the stable production of CBM wells.
  7 $$q_{wi} = \frac{{q_{wb} }}{{q_{wa} }}$$
  where, *q*_wi_ denotes the index of sudden change of daily water yield (dimensionless); *q*_wb_ and *q*_wa_ represent the daily water yields (m^3^/d) before and after the day on which the sudden change occurred, respectively.
2. Index of sudden change in daily gas production
  The index of sudden change in daily gas production refers to the ratio of the daily gas production of the previous day to that of the next day under normal drainage and production during the stable production of CBM wells.
  8 $$q_{{{\text{g}}i}} = \frac{{q_{gb} }}{{q_{ga} }}$$
  where, *q*_gi_ indicates the index of sudden change of daily gas yield (dimensionless); *q*_gb_ and *q*_ga_ represent the daily gas yields (m^3^/d) before and after the day on which the sudden change occurred, respectively.

### Determination of critical values of evaluation parameters

#### Determination of critical values of evaluation parameters for the gas production potential

**Figure 2:**
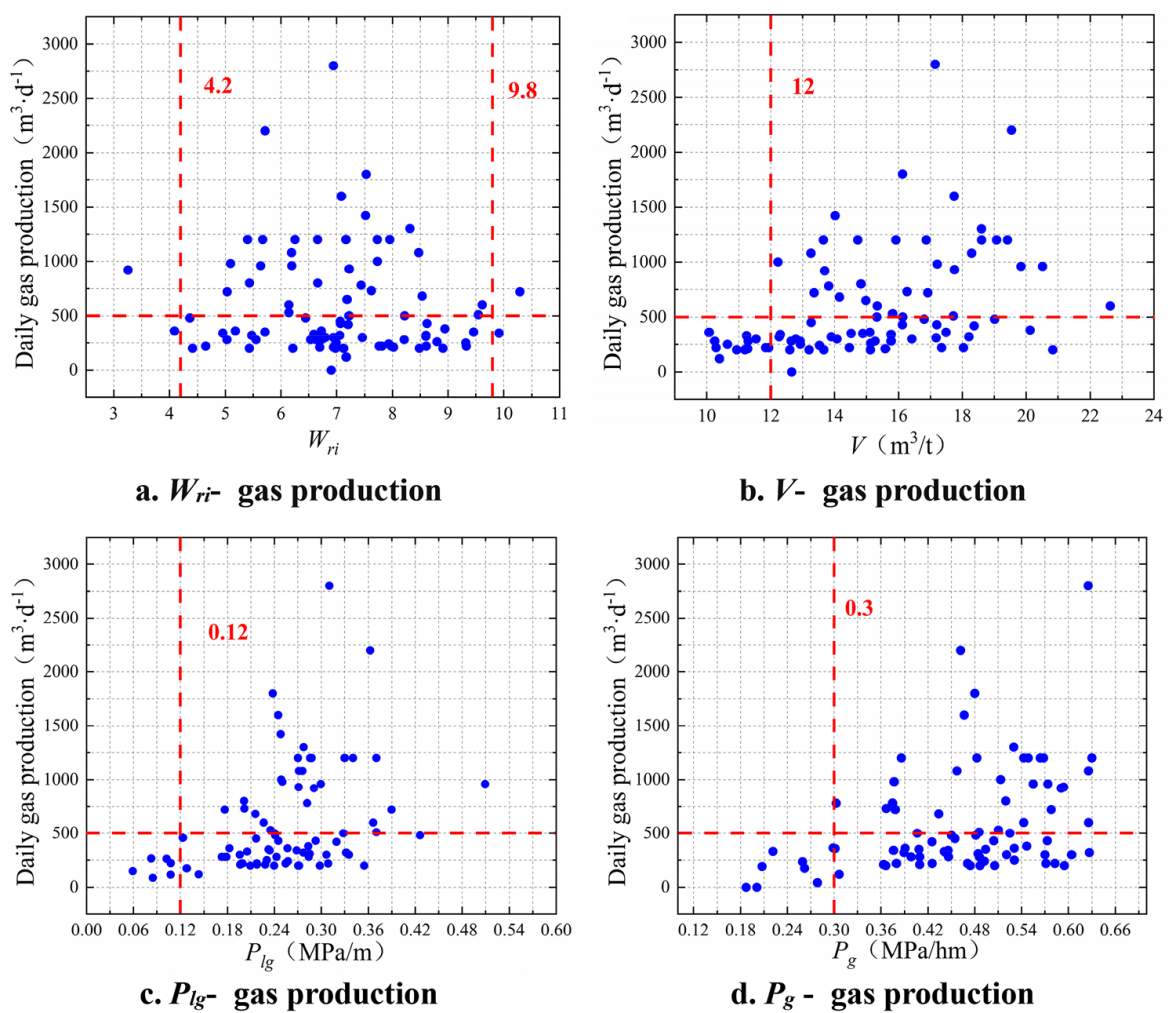
Determination of critical values of evaluation parameters for the gas production potential.

Based on production data of CBM wells in the Shizhuang South Block of Qinshui Basin, a scatter diagram of the daily water yield, gas content, desorption pressure gradient, reservoir pressure gradient, and daily gas production was plotted (Fig. 2). By taking the daily gas production of 500 m^3^/d as the boundary, the critical values of various parameters were obtained (Table 1).

**Table 1:** Determination of critical value of evaluation index.

Critical value	*W*_*ri*_	*V* (m^3^/t)	*P*_*lg*_ (MPa/m)	*P*_g_ (MPa/m)	*C*_*sr*_	*p*_*s*_	*R*_*pi*_	*q*_*wi*_	*q*_*gi*_
*MAX*	4.2	–	–	–	–	1.8	0.4	13	2.5
*MIN*	9.8	12	0.12	0.3	0.15	–	–	–	–

As shown in Fig. 2, the daily water yield index is an intermediate indicator (Fig. 2a). With the daily gas volume of 500 m^3^/d as the boundary, the daily water yield index is mainly distributed between 4.2 and 9.8 (as shown by the red dotted line). When the daily water yield index is less than 4.2 or greater than 9.8, the daily gas production of most CBM wells is less than 500 m3/d, which can be used as the critical value.

Gas content (Fig. 2b), desorption pressure gradient (Fig. 2c), and reservoir pressure gradient (Fig. 2d) are all maximum type indices: the daily gas production data are positively correlated therewith. The critical values of each parameter are 12 m^3^/t, 0.12 MPa/m, and 0.3 MPa/m, respectively. When the gas content, desorption pressure gradient, and reservoir pressure gradient exceed the corresponding critical value, it is indicative of a greater gas production potential.

#### Determination of critical values of evaluation parameters for the effective extension length of cracks

**Figure 3:**
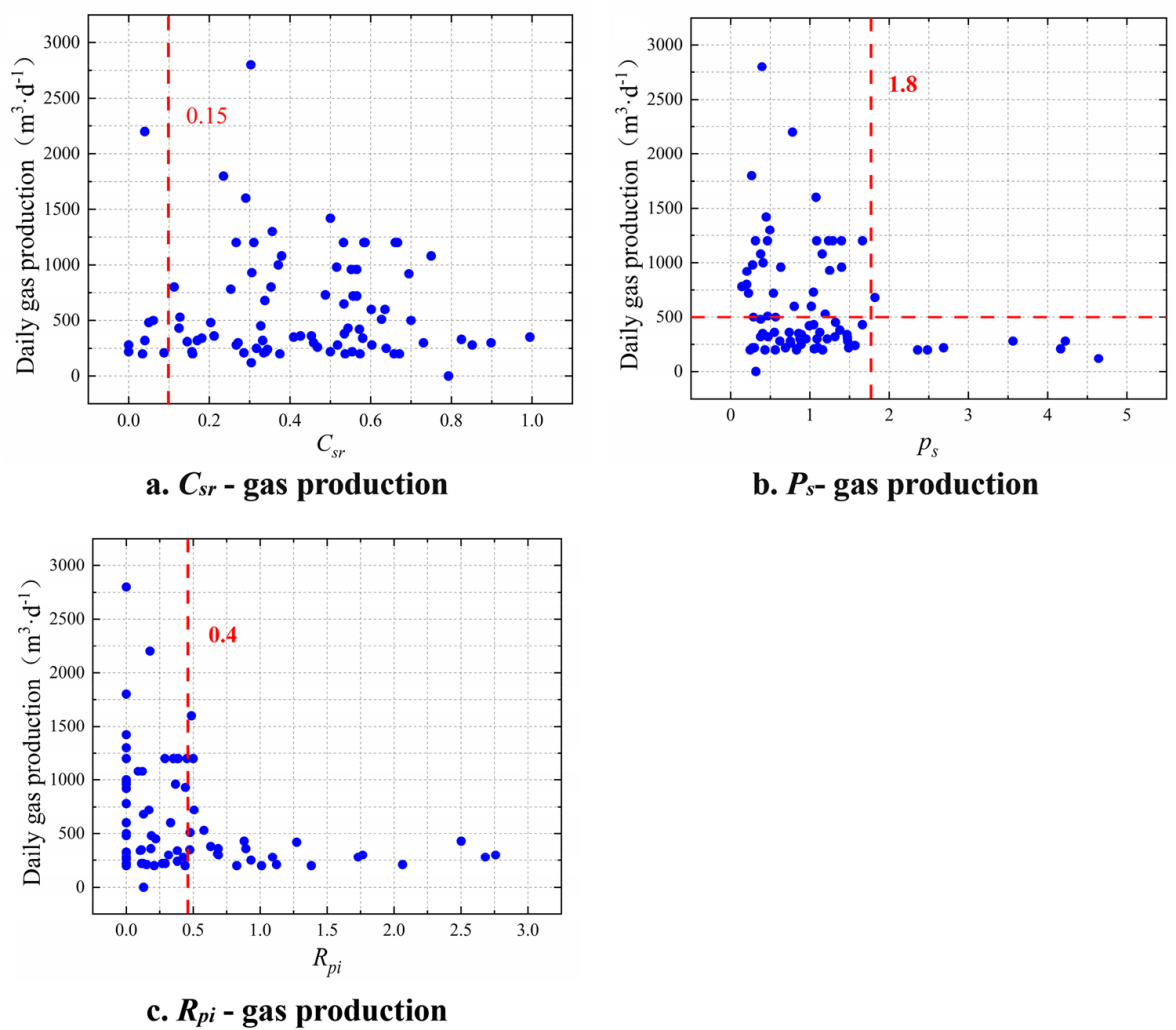
Determination of critical values of evaluation parameters for the effective extension length of cracks.

The scatter diagram of the daily gas production and effective extension length of cracks was plotted using the same method, as illustrated in Fig. 3. The critical values of various evaluation parameters were obtained (Table 1).

As shown in Fig. 3 that the thickness ratio of primary/cataclastic coal is a maximum type index (Fig. 3a), and the critical value of thickness ratio of primary/cataclastic coal is 0.15. The low proportion of primary/cataclastic coal thickness makes it difficult to produce sufficient effective fracture extension length in the reservoir, the coal reservoir cannot be effectively transformed, and the productivity of coalbed methane wells is thus low.

The construction pressure fluctuation index (Fig. 3b) and reservoir pollution index (Fig. 3c) are minimum type indices, which have a negative correlation with daily gas production. The critical values of each parameter are 1.8 and 0.4, respectively. The greater the construction pressure fluctuation index and reservoir pollution index, the lower the reservoir permeability and the lower the productivity of CBM wells.

#### Determination of critical values of evaluation parameters for the effective supporting length of proppant in cracks

**Figure 4:**
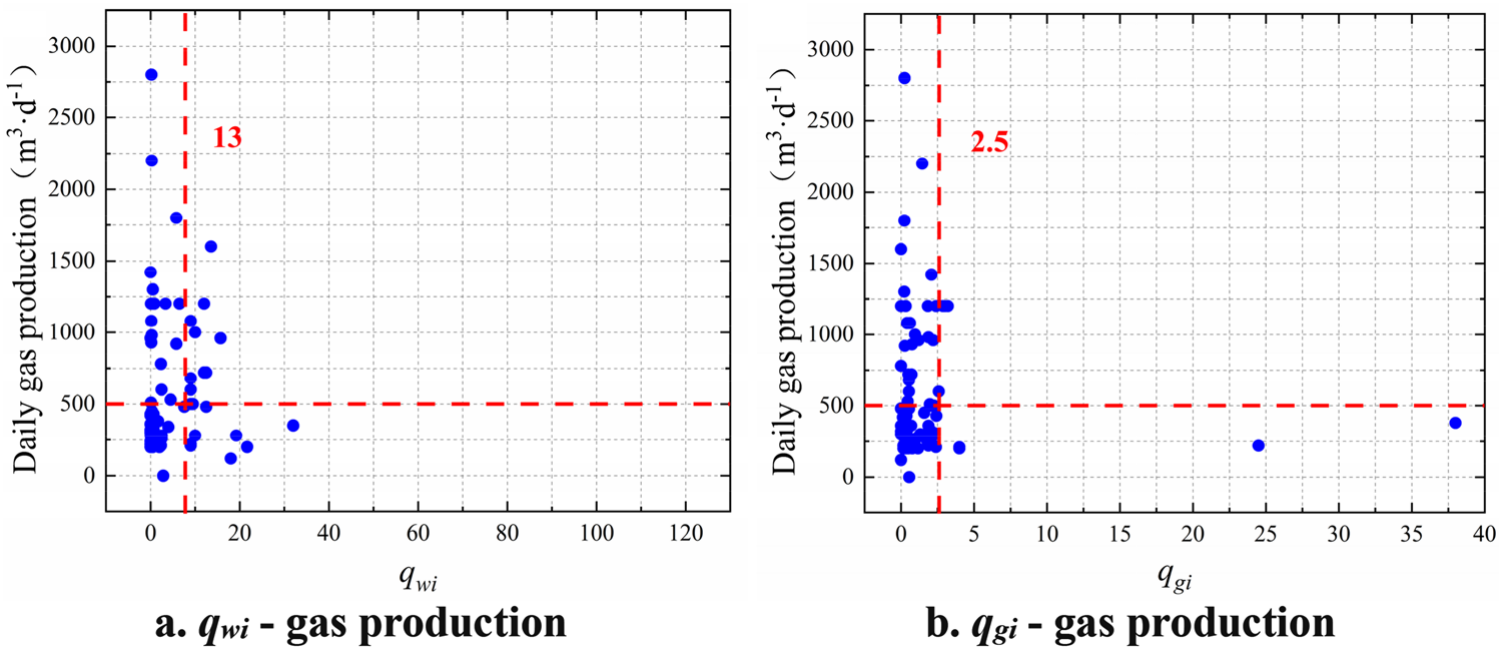
Determination of critical values of evaluation parameters for the effective supporting length of proppant in cracks.

Using the same method, the scatter diagram of the daily gas production and effective supporting length of proppant in cracks could be plotted (Fig. 4). The critical values of parameters including sudden changes of water yield and gas production were determined (Table 1).

As listed in Fig. 4, the catastrophe index of daily water production (Fig. 4a) and the catastrophe index of daily gas production (Fig. 4b) are minimum type indices, which have a negative correlation with daily gas production. The critical values of each parameter are 13 and 2.5, respectively. Changes in daily water production and gas production will affect the productivity of CBM wells. The greater the change, the lower the productivity of CBM wells.

### Weights of evaluation parameters

According to the production data of more than 100 CBM wells in the study area, the comprehensive evaluation weights of each evaluation index were obtained using PCA and EWM: in accordance with the method described in previous research^17,18^, the weights of each evaluation parameter were calculated through PCA and EWM. Furthermore, the weights obtained separately by the two methods were subjected to weighted averaging to finally identify the comprehensive weights of evaluation indices. This method can effectively avoid the subjective components arising from PCA and improve the accuracy of the evaluation^19^. The weights of each evaluation index are listed in Table 2.

**Table 2:** Evaluation index weight.

Weights	*W*_*ri*_	*V* (m^3^/t)	*P*_*lg*_ (MPa/m)	*P*_g_ (MPa/m)	*C*_*sr*_	*p*_*s*_	*R*_*pi*_	*q*_*wi*_	*q*_*gi*_
PCA	0.254	0.174	0.151	0.129	0.101	0.094	0.049	0.026	0.023
EWM	0.153	0.129	0.127	0.139	0.135	0.114	0.104	0.002	0.097
Average	0.203	0.151	0.139	0.134	0.118	0.104	0.076	0.014	0.060

The critical values of primary evaluation index are listed in Table 3.

**Table 3:** Determination of critical value of primary evaluation index.

	The gas production potential	The effective length of cracks formed by fracturing	The effective supporting length of proppant in cracks
Critical values	0.28	0.17	0.04

### Method for optimal selection of wells for secondary fracturing

**Figure 5:**
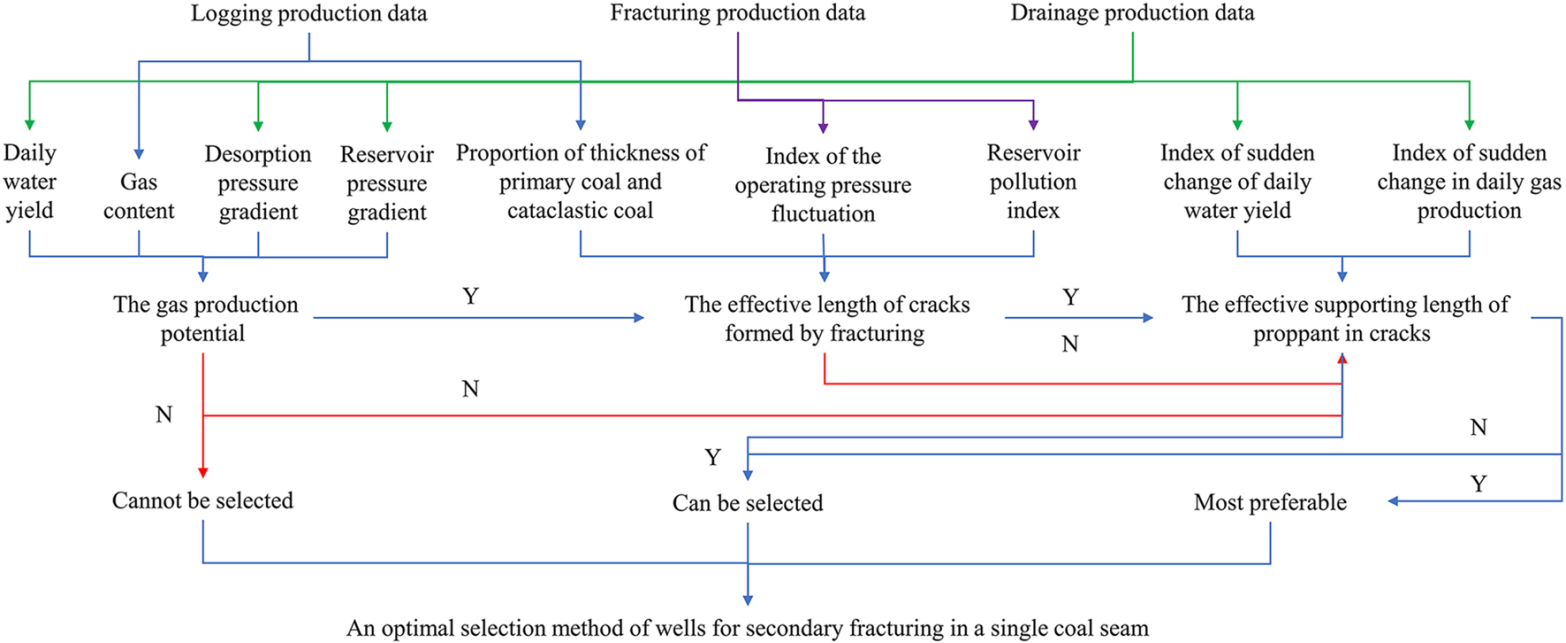
An optimal selection method of wells for secondary fracturing in a single coal seam.

After establishing the evaluation index system, the evaluation parameters could be calculated based on data of CBM wells in the study area, thus finally determining the selection of those wells deemed optimal for secondary fracturing. The specific method for optimal selection is described below (Fig. 5).

Firstly, the gas production potential is evaluated. If the evaluated value is below the critical value, the wells are deemed unsuitable for secondary fracturing. If the gas production potential conditions are met, then the effective extended length of cracks and the effective supporting length of proppant in cracks are evaluated. When the two indices evaluated are below the critical values, the corresponding wells are deemed unsuitable for secondary fracturing. When any one of the indices exceeds its critical value, secondary fracturing can be conducted. The wells for secondary fracturing can be ranked by how much their parameters exceed the critical values.

## Evaluation of the application effect

### Evaluation parameters

Eleven wells with the low production in the study area were selected for reservoir reconstruction by secondary fracturing. The evaluation parameters of the 11 wells are listed in Table 4.

**Table 4:** List of evaluation parameters of 11 low production wells.

Wells	*W*_*ri*_	*V* (m^3^/t)	*P*_*lg*_ (MPa/m)	*P*_g_ (MPa/m)	*C*_*sr*_	*p*_*s*_	*R*_*pi*_	*q*_*wi*_	*q*_*gi*_
s-01	7.76	8.95	0.23	0.43	0.28	6.30	0.10	4.40	1.05
s-02	5.13	13.81	0.13	0.21	0.81	1.23	0.00	0.67	0.08
s-03	5.64	7.78	0.15	0.39	0.62	1.50	0.41	0.23	0.24
s-04	5.00	10.31	0.18	0.33	0.70	3.77	0.09	2.50	2.00
s-05	4.25	7.66	0.09	0.32	0.63	0.74	0.00	4.00	1.15
s-06	7.01	11.13	0.16	0.48	0.10	1.69	0.08	3.00	1.27
s-07	4.28	12.30	0.21	0.34	0.36	1.62	0.31	1.80	0.75
s-08	4.48	13.32	0.30	0.41	0.73	2.38	0.46	2.33	2.00
s-09	4.92	12.50	0.23	0.32	0.47	5.53	0.28	2.14	1.16
s-10	3.36	13.94	0.18	0.42	0.32	1.31	0.20	2.09	2.13
s-11	3.30	16.42	0.39	0.52	0.92	1.45	0.31	2.15	0.14

### Optimal selection results

Based on the optimal selection results, some wells were selected for reservoir reconstruction by secondary fracturing in the field and the reconstruction effects are listed in Table 5. Among them, the average daily gas production after the transformation is the ratio of the cumulative gas production in the stable gas production period after secondary transformation to the stable production phase.

9 $$q_{gs} = \frac{{Q_{gs} }}{{T_{s} }}$$

**Table 5:** Evaluation results of whether 11 wells are suitable for secondary fracturing.

Wells	Whether the conditions of the gas production potential	Whether the conditions of the effective length of cracks formed by fracturing	Whether the conditions of the effective supporting length of proppant in cracks	Is it suitable for secondary fracturing	Average daily gas production after primary fracturing (m^3^/d)	Average daily gas production after secondary reconstruction (m^3^/d)
s-1	N	N	Y	N	80	220
s-2	N	Y	Y	N	50	95
s-3	N	Y	Y	N	21	139
s-4	N	Y	Y	N	10	78
s-5	N	Y	Y	N	10	34
s-6	N	Y	Y	N	31	198
s-7	Y	N	Y	Y	312	1006
s-8	Y	Y	Y	Y	430	533
s-9	Y	Y	Y	Y	234	360
s-10	Y	Y	N	Y	141	528
s-11	Y	Y	N	Y	362	630

where, *q*_*gs*_ denotes the average daily gas production after the transformation (m^3^/d);Qgs and Ts represent the the cumulative gas production in the stable gas production period after secondary transformation (m^3^) and Stable production time (d).

1. As listed in Table 5, in the selected 11 wells for secondary fracturing, wells s-01 to s-06 do not meet the critical value for gas production potential and have poor reconstruction effects, so they are unsuitable for secondary reconstruction. Wells s-07 to s-11 satisfy the critical value for gas production potential and exert good field reconstruction effects, so they are suitable for reservoir reconstruction through secondary fracturing.
2. Comparing the evaluation results of wells s-01, s-02, s-03, s-07, s-08, and s-09, when the evaluations of gas production potential and effective support fracture length are similar, the secondary fracturing reconstruction effect of wells with insufficient effective fracture extension length of primary fracturing is better.
3. Comparing the evaluation results of wells s-08, s-09, s-10, and s-11, under the condition that the evaluations of gas production potential and effective fracture extension length are similar, the secondary fracturing reconstruction effect of wells with low effective support fracture length is better.
4. Comparing the evaluation results of wells s-07, s-10, and s-11, when the evaluation of gas production potential is similar, the secondary fracturing effect of wells with insufficient extension length of single effective fracture is better than that of wells with low length of single effective support fracture.

In summary, when the evaluation of gas production potential is similar, the secondary fracturing wells should be implemented in order of low effective fracture extension length and effective support fracture length > low single effective fracture extension length > low single effective support fracture length > normal effective fracture extension length and effective support fracture length.

## Conclusion

1. An evaluation index system for the gas effective length of cracks formed by fracturing, and supporting length of proppant in cracks was established based on system theory. The evaluation indices were characterized through production data, such as logging, fracturing and drainage, which avoided potential bias in evaluation when only considering a certain parameter and ensured accuracy and practicability of the evaluation parameters for each well. Critical values of each evaluation parameter were determined based on actual gas production data. By using PCA and EWM, weights of evaluation parameters were determined, which could avoid the contradiction of the contributions of various parameters to the optimal selection and the rationalized results. In this way, a method for step-wise optimal selection of wells for secondary fracturing integrating construction of evaluation parameters, determination of critical values, and entropy evaluation was proposed.
2. Eleven wells with low productivity were selected in the study area for reservoir reconstruction by secondary fracturing. The results show that wells whose three evaluation indices are satisfied are most preferable; wells that only meet the criteria pertaining to the effective length of cracks formed by fracturing or effective supporting length of proppant in cracks can be selected; wells which do not meet the gas-production potential, or all of the three parameters, cannot be selected. The accuracy of the theoretical method was validated through in-situ application in practice.
3. The method of evaluation established in this study allows rapid, accurate selection of wells for secondary fracturing, which reduces the blindness encountered during such projects and improves the success rate upon construction.
